# It takes two: development of simulation training for two-handed neonatal mask ventilation integrating a respiratory function monitor; a non-randomised before-and-after simulation study

**DOI:** 10.1016/j.resplu.2026.101488

**Published:** 2026-09-14

**Authors:** Laura J. Ryan, Elizabeth Murphy, Linda Smiles, Carmel M. Moore, Mary O’Dea, Mary Higgins, Quentin Le Cornu, Anna E. Curley, Eoin M. O’Currain

**Affiliations:** a The National Maternity Hospital, Dublin, Ireland; b UCD School of Medicine, University College Dublin, Ireland

## Abstract

**Introduction:**

Facemask leak is common with a one-handed hold and can reduce the efficacy of neonatal resuscitation. Our objective was to assess a standardised, respiratory function monitor (RFM)-guided training intervention for two-handed neonatal positive pressure ventilation (PPV).

**Methods:**

Development of a two-handed PPV training algorithm occurred through an iterative process with learner and expert feedback. Participants performed PPV via a facemask on a term manikin with feedback from the RFM display (Monivent Neo100). We first piloted the training intervention on ten clinicians over six sessions. The intervention was then assessed through a before-and-after, non-randomised simulation-based study, blinded to the RFM, comparing mask leak, tidal volume and participant feedback. Mask hold style and hand preference were recorded.

**Results:**

Prior to the intervention, 17/40 (42%) had previously used two-handed PPV in clinical practice. Forty clinicians were included in the training study. Mask leak was significantly lower with a two-handed hold before training: one-handed median 27%, (IQR 3–73%) vs two-handed: 2.5% (1–31%), *p* < 0.01 median difference (95% CI) of −5.5 (−23 to −1). Post-training mask leak was comparable between holds: one-handed median 6% (IQR 2–55%); and two-handed 2% (0–5%), *p* < 0.01 median difference (95% CI) −4 (−18 to −1). Pre-training, 11/40 (27%) preferred two-handed compared with one-handed PPV. Following training 20/40 (50%) preferred two-handed.

**Conclusion:**

Effective facemask ventilation in a neonatal manikin was associated with a two-handed hold without training or prior clinical experience. After training facemask leak was low with both holds. Feedback indicated increased participant preference for two-handed PPV after training.

## Introduction

Effective facemask ventilation is an essential skill in neonatal resuscitation. Facemask leak of up to 60% in preterm neonates is common and can be associated with ineffective ventilation.^1^ In neonatal studies of mask leak, good technique, rather than mask shape or size, appears to have the best association with leak reduction.^2^ Manikin-based studies have demonstrated reduced leak with two-handed positive pressure ventilation (PPV) compared with one-handed.^3^, ^4^ A clinical pilot study of 25 preterm neonates demonstrated lower leak with two-handed PPV in the delivery room.^5^ The Neonatal Resuscitation Programme (NRP) includes consideration of use of a two-handed hold during mask readjustment,^6^ but there is no specific requirement to learn nor demonstrate a two-handed hold for NRP certification. Respiratory function monitors (RFM) have been established as useful training adjuncts in neonatal facemask ventilation, with reduced leak demonstrated following RFM-based manikin educational interventions.^7^, ^8^ The simulated environment allows for examination of this rare event in a standardised fashion.

## Objective

To assess a standardised training intervention in two-handed neonatal facemask ventilation, guided by a respiratory function monitor (RFM), in a before-and-after simulation-based study.

## Methods

This is a non-randomised, before-and-after simulation-based study, reported in accordance with the STROBE statement, with extensions for simulation-based research^9^ (see supplementary material). This study was approved by the Research Ethics Committee of the National Maternity Hospital, Dublin, Ireland (EC 36 2024). We obtained prospective informed consent from participants.

### Development

Five subject matter experts (SMEs) in neonatology, nursing and simulation-based education formed an expert group and designed the training using an adapted simulation design framework, aligned with best practice.^6^, ^10^, ^11^, ^12^ A needs assessment identified that a previously developed RFM-based standardised training algorithm for neonatal facemask ventilation^10^ lacked specific instruction in two-handed PPV. Measurable objectives, including mask leak reduction and optimisation of tidal volume (TV) delivery, were identified. Learning objectives included interpretation of feedback and improved ability to recognise and correct suboptimal technique.^10^

A simulation-based scenario was created by the group. The scenario was piloted and developed through an iterative process over six sessions (Table 1). A standardised, reproducible training algorithm (Fig. 1) was created, with the goal of achieving mask leak of <30% and TV of 4–6 ml/kg, allowing for standardisation of training across instructors. We utilised a procedural simulation platform to facilitate deliberate practice, after a scripted demonstration by the instructor. A pre-brief was developed to provide expected learning objectives. To maintain fidelity, all sessions occurred *in-situ* in the neonatal intensive care unit (NICU), using a Neopuff Infant T-piece Resuscitator (Fisher & Paykel) and a standardised Fischer & Paykel facemask. A term manikin was adapted to make it ‘leak-free’ with a 50 ml test lung (Dräger, Lubeck, Germany).^13^ The Monivent Neo100 RFM (Göteborg, Sweden) was chosen for its user-friendly display. A learner-focused facilitative approach was cultivated by establishing a psychologically safe environment, with sessions tailored to the learner’s individual needs. During the period of deliberate practice, learners were encouraged to interrogate RFM feedback and correct suboptimal technique. If required, instructors would help adjust technique and provide training, as indicated as per algorithm. After training, feedback and evaluation were provided via debriefing and post-training surveys, developed following the Kirkpatrick evaluation model.^14^ Objective comparison data were collected, through before-and-after PPV assessments.

**Table 1:** Iterative development of training tool.

**Training session**	**Change**	**Details**
1	Survey development	Development of survey to include learners’ prior experience with a two-handed hold, their preferred hold, hand preference for one-handed, glove size, simplified and rationalised questions to maximise information
2	Individualised learning	One-to-one training sessions Removed one-handed reassessment if mask leak was low during pre-training assessment Removed routine description of the various holds, described only if learner appeared to be uncomfortable with their current hold
3	Utilisation of supportive educational resources	Addition of visual aids with photographs of different hold styles
4	Optimised training efficiency	Shortened pre-brief Shortened instructor demonstration, increased efficiency and made more learner-focused Addition of focus on team communication to optimise rate/timing of delivered breaths, hand position
5	Increased feedback potential	Changed to online version of feedback survey with participant number to ensure candour
6	Improved data collection	Respiratory Function Monitor: Assigned each participant one file to ensure accurate data collection Rationalised study case report form to ensure accurate data entry

**Fig. 1:**
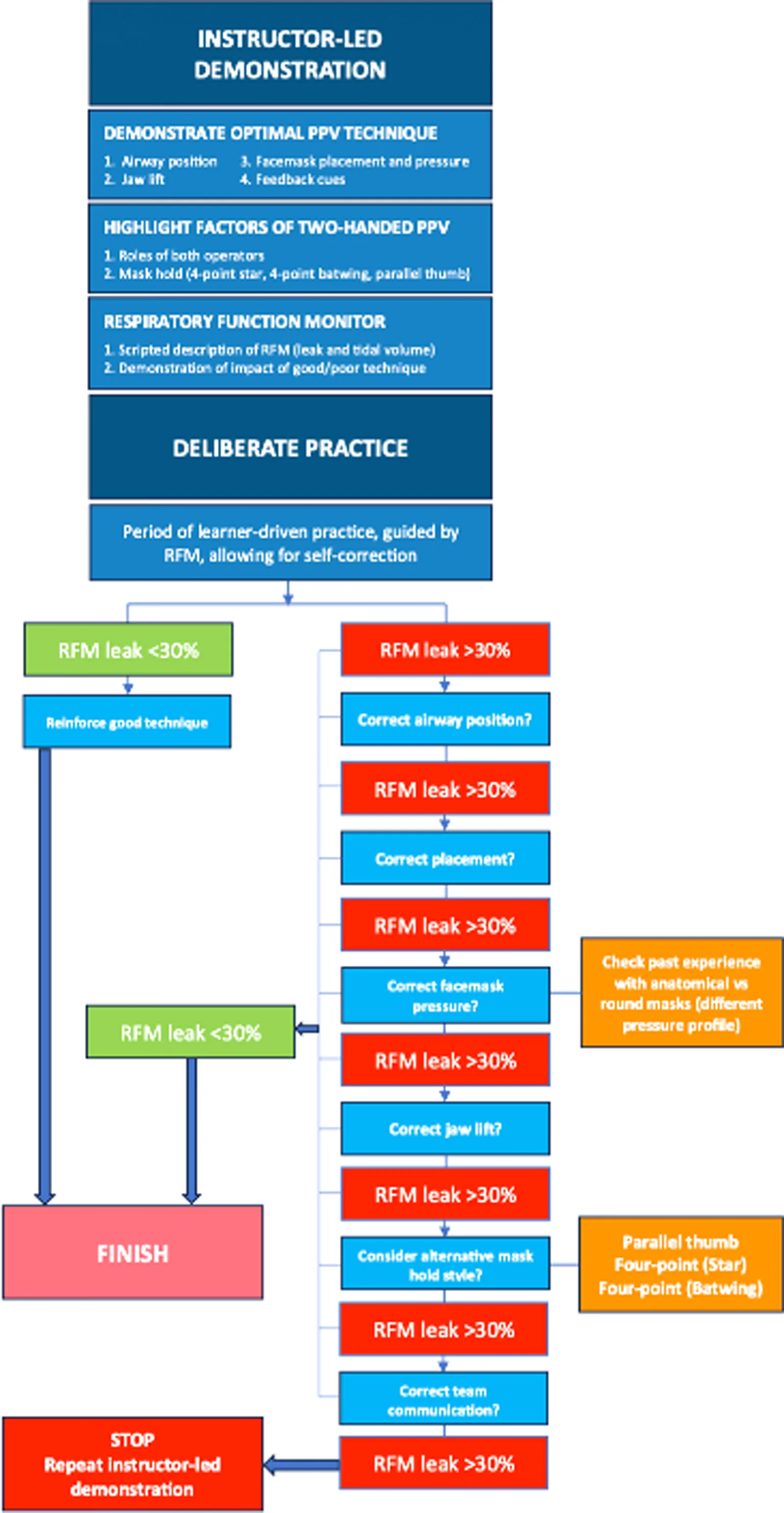
**Training algorithm: two-handed facemask ventilation training (RFM = Respiratory Function Monitor)**.

### Training assessment

NRP certified NICU doctors and nurses were eligible for recruitment. PPV was delivered via a standardised facemask and T-piece Resuscitator to a term manikin simulating an apnoeic infant^13^ in a tertiary NICU.

Pre-training, all participants performed 30 s of unguided one and two-handed facemask ventilation, in that order. The RFM display was blinded to the learner but visible to the instructor. If one-handed technique was poor (leak > 30%), standardised RFM-guided training was administered for both one and two-handed PPV^11^ (Fig. 1). If one-handed technique was adequate (leak < 30%), the learner progressed to two-handed RFM-guided training. After training, all participants performed a further 30 s of one and two-handed PPV blinded to the RFM, in that order. The primary outcome was mask leak. TV, style of mask hold, technique assessment and feedback surveys were also recorded for each participant.

### Statistical analysis

This was a convenience sample, which represented 69% of eligible NICU staff on the days when the study took place. No formal sample size calculation nor further considerations were undertaken.

Descriptive statistics summarised continuous variables as mean (standard deviation) if parametric distribution and median (interquartile range) if non-parametric and categorical variables as frequencies (percentages). No missing data were observed in the dataset. Inferential statistics were used to compare mask leak between hold techniques (one-handed vs. two-handed) and before and after training.

We summarised repeated leak measures for each participant using the median and compare pre- and post-training medians using a Wilcoxon signed-rank test. This approach simplified the statistical analysis and interpretation but does not account for the within-participant variability. Therefore, we conducted a mixed-effect quantile regression analysis with bootstrapped standard errors and estimated median difference with 95% confidence intervals. Survey data were summarised using descriptive statistics or thematic analysis, as appropriate.

All analyses were conducted using Stata version 19.5 (significance level of 0.05).

## Results

Forty participants were recruited to undertake the finalised training programme (Table 2). One participant also contributed to the iterative development. Mean training duration was 7 min 8 s (Table 3). Mask leak was lower in two-handed PPV in pre and post-training groups. Pre-training mask leak: one-handed median 27%, (IQR 3–73%) vs two-handed median 2.5% (1–31%), *p* < 0.01 with median difference in leak (95% CI) −5.5 (−23 to −1). Post-training mask leak: one-handed median 6% (IQR 2–55%) vs two-handed median 2% (0–5%), *p* < 0.01 with median difference in leak (95% CI) −4 (−18 to −1) (see Table 3).

**Table 2:** Demographics of participants in training.

**Characteristic**	**Total (*n* = 40)**
**Professional role, *n* (%)**	
Paediatric trainees	18 (45)
Neonatal fellows	3 (8)
Consultants	5 (12)
Neonatal staff nurses	6 (15)
Neonatal specialist nurses	7 (18)
Advanced Nurse Practitioner	1 (2)
**Neonatal Intensive Care Unit experience, *n* (%)**	
<1 year	13 (33)
1–5 years	9 (22)
5–10 years	4 (10)
>10 years	14 (35)
**Gender/Sex, *n* (%)**	
Female	30 (75)
Male	9 (23)
Non-binary	1 (2)
**Neonatal Resuscitation Programme certification, *n* (%)**	
Yes	40 (100)
No	0 (0)

**Table 3:** Respiratory parameters, mask hold and training duration.

	**Pre-training (*n* = 40)**		**Post-training (*n* = 40)**	
**Positive pressure ventilation**	**Two-handed**	**One-handed**	**Two-handed**	**One-handed**
Mask leak (%) median (IQR)	2.5 (1–32)	27 (3–74)	2 (0–5)	6 (2–56)
Tidal volume (ml/kg) median (IQR)	5.5 (4.9–5.9)	4.9 (3.3–5.9)	5.5 (5–6)	5.3 (3.9–6)
Peak inspiratory pressure (cmH_2_O) median (IQR)	21 (20.2–21.4)	20.4 (19.9–21)	20.7 (20.1–21.3)	20.5 (19.9–21.2)
Respiratory rate	51 (49–54)	43 (39–49)	54 (51–56)	45 (39–51)
**Style of two-handed hold, *n* (%)**				
Four-point (two-handed version of two-point)	23 (58)	n/a	17 (42)	n/a
Batwing (straight index fingers and thumbs held in an inverted triangle across the mask)	14 (36)		11 (28)	
Parallel Ski (thumbs held straight across mask in parallel, with fingers providing jaw support)	1 (2)		11 (28)	
Double OK Rim	1 (2)		1 (2)	
Three-point	1 (2)		0 (0)	
**Style of one-handed hold, *n* (%)**				
Two-point	n/a	27 (68)	n/a	28 (70)
OK Rim		12 (30)		11 (28)
Spider		0 (0)		0 (0)
Stem		1 (2)		1 (2)
**Duration of training, mean (SD)**				
All sessions	7 min 08 s (2 min 11 s)			
Senior House Officer	7 min 44 s (2 min 24 s)			
Registrar	7 min 29 s (1 min 29 s)			
Neonatal Fellow	4 min 22 s (1 min 10 s)			
Nurse	8 min 00 s (2 min 00 s)			
Consultant	4 min 47 s (1 min 00 s)			

Nineteen of 40 participants (47%) had prior training in two-handed PPV and 17/40 (42%) had used it in clinical practice. Prior to training, 11/40 (28%) preferred a two-handed hold, 26/40 (65%) preferred one-handed and 3/40 (7%) had no preference. Post-training, preference for two-handed increased to 20/40 (50%), with 15/40 (37%) preferring one-handed and 5/40 (13%) reporting no preference. Preference for hold style within one-handed and two-handed PPV varied (Table 3), including ‘four-point’ (two-handed version of two-point), ‘batwing’ (straight index fingers and thumbs held in an inverted triangle across the mask) and ‘parallel thumb’ (thumbs held straight across mask in parallel, with fingers providing jaw support). Technique corrections during training included head position (*n* = 2), jaw thrust (*n* = 8), facemask placement (*n* = 9), hand position (*n* = 14), applied mask pressure (*n* = 21) and change of hold style (*n* = 14). Twenty-nine of 40 participants (73%) felt that their technique had improved subjectively post training. Several participants reported having to concentrate less during two-handed PPV. One participant suggested the reduced ‘cognitive load’ of two-handed PPV might allow for improved situational awareness during a resuscitation. Thirty-nine of 40 participants (98%) found the training extremely or very useful.

## Discussion

Current practice is to use a one-handed hold for neonatal mask ventilation, with a two-handed hold considered only when facemask ventilation is ineffective.^6^, ^12^ Fewer than half of the participants in our study had received any formal training in two-handed mask ventilation. We developed a standardised intervention to address training in this area, and our short, focused training was associated with improved skill acquisition and reduced mask leak. In this simulated setting, a two-handed hold was associated with lower mask leak prior to training. After training, facemask leak was comparable between one and two-handed holds. This study highlights that, without recent formalised training, effective facemask ventilation in a manikin was more reliably associated with a two-handed hold.

While most participants preferred one-handed hold prior to training, preference for the two-handed hold increased post-training. Participant feedback also suggested an association between two-handed ventilation and reduced cognitive load, which may allow for better situational awareness in a real-life resuscitation. Eye-tracking has been used in manikin studies to assess participant attention, with differing visual gazes demonstrated between mask holds.^15^, ^16^ Assessment of the team leader performing mask ventilation could further investigate the association between cognitive load and the two-handed hold.

Our study was strengthened by neonatal and simulation expert review, with multiple iterations, to develop a high-quality educational intervention that may be tailored to all learners, and feasible to use in a busy neonatal unit. Our study is limited by its design as a non-randomised before-and-after manikin simulation-based study, which cannot directly replicate clinical scenarios and may not translate to clinical practice. As a single tertiary centre study, we have not tested its generalisability, nor have we assessed skill retention. The need for an additional team member for the two-handed technique is a limitation in clinical practice.

Future clinical testing may inform how the holds compare *in-vivo* and simulation-based research may assess skill retention over time and without RFM availability.

## Conclusion

In our single group before-and-after simulation-based study, the two-handed hold was associated with lower mask leak compared with one-handed, even without formal training or prior clinical experience. After training, facemask leak was low in both holds, though two-handed remained the lower of the two. Feedback indicated increased participant preference for two-handed mask hold after training.

## CRediT authorship contribution statement

**Laura J. Ryan:** Writing – review & editing, Writing – original draft, Methodology, Investigation, Formal analysis, Data curation, Conceptualization. **Elizabeth Murphy:** Writing – review & editing, Methodology, Investigation. **Linda Smiles:** Writing – review & editing, Investigation, Data curation. **Carmel M. Moore:** Resources, Funding acquisition. **Mary O’Dea:** . **Mary Higgins:** Writing – review & editing, Supervision. **Quentin Le Cornu:** Writing – review & editing, Methodology, Formal analysis. **Anna E. Curley:** Writing – review & editing, Supervision, Methodology, Formal analysis, Data curation, Conceptualization. **Eoin M. O’Currain:** Writing – review & editing, Supervision, Methodology, Formal analysis, Data curation, Conceptualization.

## Declaration of competing interest

The authors declare that they have no known competing financial interests or personal relationships that may have influenced the work reported in this paper.

## Appendix A Supplementary material

Supplementary Data 1 STROBE Checklist for simulation-based education.
