# Supplementary material for: It takes two: development of simulation training for two-handed neonatal mask ventilation integrating a respiratory function monitor; a non-randomised before-and-after simulation study
Source: Resusc Plus. 2026 Sep 14;32:101488. doi: 10.1016/j.resplu.2026.101488 (PMC13634061; doi:10.1016/j.resplu.2026.101488)
Supplement: Supplementary file 1 [file mmc1.docx]

STROBE Checklist:

Item 1 (title/abstract): It has been clearly stated in the title and abstract that this is a simulation-based study.

Item 2 (background): Justify the rationale for using simulation as the study methodology. It is outlined in the introduction that simulated events allow for examination of this rare event in a standardised fashion which maintains patient safety.

Item 7 (variables): The equipment and manikin used are stated in the methods; the location and simulated event are stated; standardised environment to minimise confounding variables.

Item 8 (data sources/measurement): The RFM collected the respiratory outcome data, and the researchers collected participant feedback.

Item 12 (statistical methods): We summarised repeated leak measures for each participant using the median and compare pre- and post-training medians using a Wilcoxon signed-rank test. This approach simplified the statistical analysis and interpretation but does not account for the within-participant variability. Therefore, we also conducted a mixed-effect quantile regression analysis with bootstrapped standard errors to estimate effect size with 95% confidence intervals.

Item 14 & 16 (descriptive data & main results): it is stated that the findings are from a simulated environment.

Item 19 (limitations): limitations of simulation-based research are clearly stated

Item 21 (generalisability): potential for generalisability has been outlined, as well as the limitation of this being a single-centre study which has not tested the generalisability outside of this setting.

Item 22 (funding): it is stated that there are no conflicts of interest and that funding received for this project is not related to the simulator brand/manufacturer.
